# The Spectrum of Differences between Childhood and Adulthood Celiac Disease

**DOI:** 10.3390/nu7105426

**Published:** 2015-10-22

**Authors:** Rachele Ciccocioppo, Peter Kruzliak, Giuseppina C. Cangemi, Miroslav Pohanka, Elena Betti, Eugenia Lauret, Luis Rodrigo

**Affiliations:** 1 Rachele Ciccocioppo, Center for the Study and Cure of Celiac Disease, Clinica Medica I, Department of Internal Medicine, IRCCS Policlinico San Matteo Foundation, University of Pavia, 19–27100 Pavia, Italy; cangemi.giusy@gmail.com (G.C.C.); elena.betti19@gmail.com (E.B.); 2 International Clinical Research Center, St. Anne’s University Hospital and Masaryk University, 65691 Brno, Czech Republic; peter.kruzliak@savba.sk; 3 Faculty of Military Health Sciences, University of Defence, Trebešská 1575-500 01 Hradec Kralove, Czech Republic; miroslav.pohanka@gmail.com; 4 Department of Geology and Pedology, Faculty of Forestry and Wood Technology, Mendel University in Brno, 61300 Brno, Czech Republic; 5 Gastroenterology Unit, Hospital Universitario Central de Asturias, 33000 Oviedo, Spain; meugelb@hotmail.com (E.L.); lrodrigosaez@gmail.com (L.R.)

**Keywords:** adulthood, associated diseases, childhood, complications

## Abstract

An old saying states that ‘’children are not little adults” and this certainly holds true for celiac disease, as there are many peculiar aspects regarding its epidemiology, diagnosis, clinical presentations, associated diseases, and response to treatment in pediatric compared to adult populations, to such an extent that it merits a description of its own. In fact, contrary to the past when it was thought that celiac disease was a disorder predominantly affecting childhood and characterized by a malabsorption syndrome, nowadays it is well recognized that it affects also adult and elderly people with an impressive variability of clinical presentation. In general, the clinical guidelines for diagnosis recommend starting with specific serologic testing in all suspected subjects, including those suffering from extraintestinal related conditions, and performing upper endoscopy with appropriate biopsy sampling of duodenal mucosa in case of positivity. The latter may be omitted in young patients showing high titers of anti-transglutaminase antibodies. The subsequent management of a celiac patient differs substantially depending on the age at diagnosis and should be based on the important consideration that this is a lifelong condition.

## 1. Introduction

Celiac disease (CD) is a chronic, immune-mediated enteropathy caused by the ingestion of gluten-containing cereals (wheat, rye, and barley) in genetically predisposed individuals [1,2]. Left untreated, CD may cause malabsorption [3], reduced quality of life [4], iron deficiency [5], osteoporosis [6,7], and an increased risk of lymphoma [8,9]. CD patients may complain of not only gastrointestinal symptoms, but also extraintestinal symptoms and, most importantly, they may often be asymptomatic [10]. Moreover, CD is associated with several autoimmune diseases [11], mostly diabetes mellitus type 1 [12] and thyroiditis [13]. The ubiquitous transglutaminase type 2 (TG2) enzyme plays a crucial role in CD pathogenesis, not only because it is the main autoantigen and the target of the specific auto-antibodies (TG2A), but also for the ordered and precise deamidation of gliadins that generates epitopes which bind more efficiently to histocompatibility locus antigen (HLA)-DQ2/DQ8 molecules [14]. Mucosal CD4^+^ T lymphocytes, in turn, become activated by these immunodominat epitopes, thus resulting in the development of an immune reaction where interleukin (IL)-15 [14,15] and interferon-α are involved [16]. The TG2A are produced by B-cells localized in the intestinal mucosa, and detected in patients’ serum. Moreover, TG2 activity may lead to unmasking of further auto-antigens thus causing additional autoimmune conditions [17]. The activation of these complex pathogenic mechanisms leads to various degrees of alteration of small intestine architecture eventually resulting in malfunction of the intestine [18]. Currently, the only effective treatment available is a strict life-long gluten-free diet (GFD), which improves symptoms, nutritional status [19,20], serological and histological changes [19], and possibly body composition in most patients [19,20,21].

## 2. Epidemiology and Genetics

It is estimated that CD affects approximately 1% of the whole world population [22]. Recent epidemiological research based on serological studies of the general population in Europe and in the United States of America has also shown that both the incidence and prevalence has grown in recent years not only thanks to increased awareness and better diagnostic tests, but also due to a real spread of the disease [22]. Scandinavian, Irish and United Kingdom populations tended to show a higher prevalence of CD compared to the rest of Europe [23]. On the other hand, there are regions with very low or no incidence of CD North, Sub-and Saharan Africa, India, China, Japan, and the Caribbean [24,25]. The low incidence is probably due in part to eating habits, but genetic disposition may also play a role. Unavailability of proper diagnostic facilities can be another reason why incidence appears to be so low in these regions [25]. With regard to gender, there is an increased prevalence of CD amongst women compared to men with a male: female ratio of 1:2.8 [26]. However, a gap has emerged between the prevalence rate obtained through serological screening and that resulting from clinical studies. This is due to the fact that oligo-a-symptomatic patients or those with atypical forms too often remain unrevealed [24]. In fact, the ratio between diagnosed and undiagnosed cases was as high as 1 to 7 (the “celiac iceberg”) [27]; for instance, the Danish National Patient Registry records about 50 cases of patients per 100,000 persons [28].

As far as genetic predisposition is concerned, it is estimated that almost all CD patients carry the HLA-DQ2 or HLA-DQ8 haplotype (with HLA-DQ2 prevalence being >90% and HLA-DQ8 approximately 5%) [29]. The molecules encoded by these genes play a key role in the pathogenesis of the disease since they form a heterodimer expressed on the surface of antigen-presenting cells which displays high affinity to gliadin peptides, thus triggering and sustaining the abnormal adaptive immune response to these epitopes [30]. Nevertheless, HLA-DQ2 and HLA-DQ8 occur in about 30% of the Caucasian population, thus HLA genotyping did not increase the diagnostic accuracy of the serological tests, since a positive result has low specificity [31]. On the other hand, it has a strong negative predictive value, since the absence of HLA-DQ2 and HLA-DQ8 can most likely exclude CD. Thus, HLA-DQ2/8 genotyping can be used as complementary analysis in some specific situations, e.g., in patients with a discrepancy between serology and histology, and where complications are suspected [32]. Further genetic *loci* have been shown to be related to CD susceptibility and possibly also to its pathogenesis [33]. The most important ones are the following: CELIAC1 on chromosome 6, CELIAC2 on chromosome 5q31-33 [34], CELIAC3 on chromosome 2q33 [35], and CELIAC4 on chromosome 19p13.1 [36].

## 3. Serologic and Histologic Differences between CD in Children and Adults

The diagnosis of CD has traditionally depended upon the results of several (four to six) intestinal biopsies, currently considered to be the gold standard, and has been extended to include also an array of serological markers.

The Guidelines of the North American Societies for Gastroenterology usually require at least a duodenal biopsy for diagnosis [37,38]. Recently, the European Society for Pediatric Gastroenterology, Hepatology, and Nutrition (ESPGHAN) published new guidelines allowing the diagnosis of CD without a biopsy in some situations, mainly related to presence of high titers of TG2A, higher than 100 IU/L [39]. CD is usually diagnosed when the duodenal or jejunal mucosa displays changes including not only a different degree of villous atrophy but also crypt hyperplasia, and an increase in intraepithelial lymphocytes (IELs) [40].

However, other diseases unrelated to gluten-dependent enteropathy can induce a flat mucosa, thus mimicking CD (see below), whilst CD may exist even in the presence of a normal small bowel mucosal architecture [41]. In addition, often the lack of technical proficiency in using biopsy forceps and/or of orientation of the mucosal specimens are the cause of perplexing interpretations of the original histologic preparations, indeed they have been shown to be sufficient for CD diagnosis in only 90% of cases [42].

Furthermore, CD may be overlooked during histological examinations, owing to differences in pathologists’ assessments. Because of this, and also due to the inconvenience and the cost associated with jejunal biopsy and the high prevalence of CD in the general population, less-invasive tests are required. Over the last 20 years, several new serological tests have been used for the diagnosis of CD, leading to a significant improvement in accuracy. For practical and ethical reasons, patients with negative serology sometimes do not undergo a biopsy unless clinical indications of CD are evident (IgA deficiency). This procedure causes a verification bias because the gold standard (histology of the mucosa) is not always available for negative tests [43]

A positive correlation between serum levels of TG2A and duodenal histopathology has been previously described, both for pediatric and adult CD populations [44,45]. In a prospective clinical study, when comparing the findings of TG2A in these two populations, a positive correlation between the TG2A titers and the mucosal lesions according to Marsh grades was consistently observed in the pediatric population [46].

Although TG2A levels correlated with duodenal histopathology both in the adult [47] and pediatric populations [46], the higher percentage of Marsh type 3 lesions observed in children [48] makes a high antibody titer especially interesting for CD prediction in this group. The choice of an upper cut-off limit of 30 U/mL TG2A yielded the highest area under the receiver operating characteristic curve (0.854). Based on the predictive value of this cut-off point, up to 95% of children and only 53% of adults would be correctly diagnosed without biopsy. Thus, the authors conclude that duodenal biopsy may be avoided when high TG2A titers are present, generally over 100 IU/mL [46].

**Figure 1:**
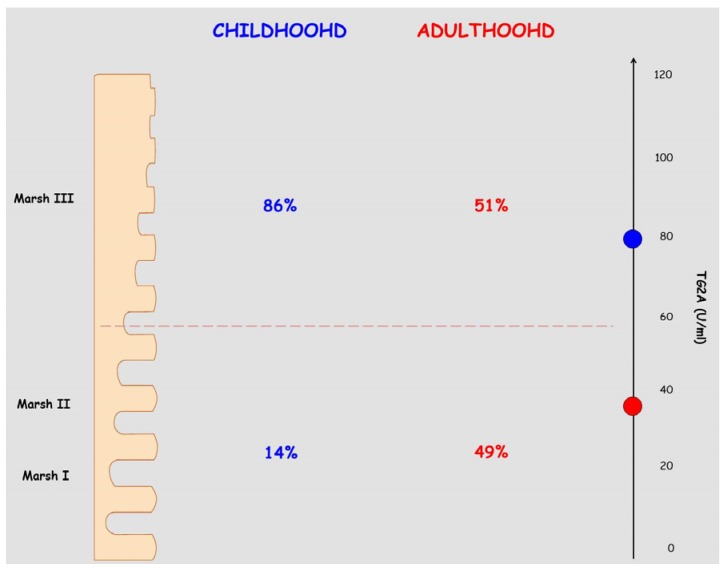
Serologic and histologic findings in childhood and adulthood celiac disease. The prevalence of major and minor intestinal lesions, as defined according to Marsh’s classification, in terms of percentages in both childhood and adult celiac disease is shown. Moreover, the mean values of anti-tissue transglutaminase antibodies (TG2A) is given on the right hand side, with the blue spot indicating childhood age and the red one adult age. The data are presented according to [50].

Several additional studies in extensive series of celiac patients have clearly shown that TG2A sensitivity varies depending on the severity of duodenal damage, and reaches almost 100% in the presence of complete villous atrophy (more common in children under three years), 70% for subtotal atrophy, and up to 30% when only an increase in IELs is present [49]. However, TG2A titers and histologic lesion degree exhibit an inverse correlation with age [50]. Thus, as the age of diagnosis increases, the antibody titers decrease and histological damage is less marked. It is common to find adults without villous atrophy, showing only an inflammatory pattern in the duodenal mucosa biopsies, *i.e.*, increased number of IELs (Marsh I) with/without crypt hyperplasia (Marsh II) (Figure 1) [50].

However, a note of caution should be inserted when diagnosing CD in children younger than two years of age, since the biopsies before and after the gluten challenge may be omitted only in the presence of anti-endomysial antibodies (EMA) positivity and Marsh IIIc lesions at histology [48].

## 4. Associated Diseases

**Figure 2:**
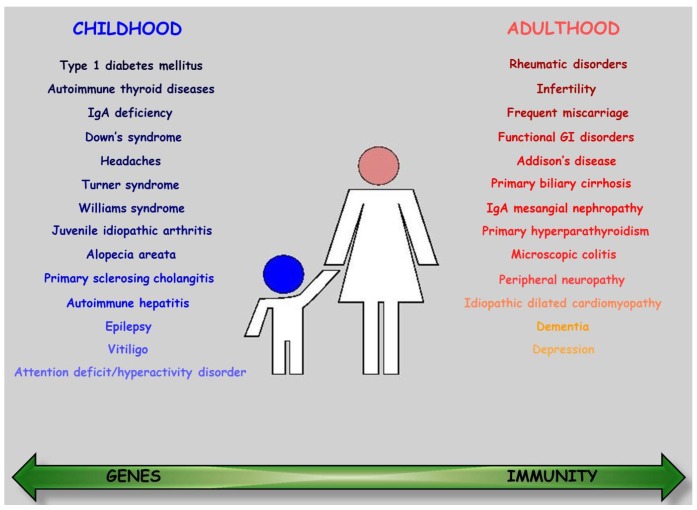
Associated diseases. The most common associated diseases listed in descending order of frequency for childhood (**left**) and adulthood (**right**) celiac disease. The green arrow shows the prevalent role played by genes or immunity in the pathogenesis of these conditions. Abbreviations: GI: gastrointestinal; Ig: immunoglobulin.

The number of conditions possibly associated with CD is extensive enough to justify active screening for most of them, with an estimated prevalence of 30.1% in adulthood and 20.7% in childhood [51]. The most frequent are the autoimmune disorders, both systemic and organ-specific, which tend to occur more frequently as the age at diagnosis increases, which can be taken as an index of the duration of gluten exposure [52]. In this regard, the protective role of the GFD was highlighted in a study where those strictly adherent to a GFD were found to acquire fewer autoimmune diseases than those not compliant with the strict dietetic regimen [53]. However, there is significant controversy surrounding the notion that early detection and treatment of CD may result in a decreased risk of developing further autoimmune diseases [54,55]. It is conceivable that, similarly to inflammatory bowel disease [56], the onset and activity of some of them are connected to the full-blown status and duration of the intestinal disease, whilst others are completely unrelated. The exact nature of this association is still not fully understood [57]. Predominant and not mutually exclusive hypotheses include the presence of a linkage disequilibrium of genes that generally predispose to autoimmune diseases [58], the loss of intestinal barrier integrity [59], an altered microbiome [60], and posttranslational modifications of immunogenic peptides [61], with a conceivable high relevance of genetics in childhood and immunity in adulthood (Figure 2). From a clinical point of view, the relevance of this association regards either the detrimental effect that an autoimmune disease may have on CD (and *vice versa*), or the possibility of diagnosing those CD patients presenting only or apparently with symptoms of secondary autoimmunity. In fact, in a substantial number of cases, both in adults and children, the disease remains clinically silent and the only manifestation is the associated disease/s [62,63] for which they are referred to specialists other than gastroenterologists, *i.e.*, endocrinologists, rheumatologists, orthopedists *etc.*, who need to be aware of this association [64]. Here below, we review the spectrum of associated diseases in CD and discuss their possible pathophysiologic mechanisms (Figure 2).

### 4.1. Type 1 Diabetes Mellitus

Surely, diabetes mellitus type I is the most frequent autoimmune disease associated with CD [65], with a prevalence of CD in type 1 diabetes that is higher in children (6.2%) than in adults (2.7%) [66]. In the former, the risk of having both the diseases is three times higher in those diagnosed with type 1 diabetes aged <4 years than in those diagnosed aged >9 years [67]. Moreover, type 1 diabetes is only seldom found in individuals already diagnosed with CD, mostly within five years of CD diagnosis and before 20 years of age [68], while in the vast majority of cases, CD is detected via screening antibodies at the time of or after the diagnosis of diabetes [69]. The need for CD screening is also justified by the evidence of potential improvement in symptoms (if present) [70], body composition [70,71,72], diabetic control [71,72], and reduction in hypoglycemia episodes after diagnosis of CD and implementation of a GFD [73], despite the higher glycemic index and fat content of gluten-free foods compared to their gluten-containing counterparts [74]. As regards the pathogenic mechanisms, other than a shared genetic background, namely the HLA genotype DR3-DQ2, gluten seems to be involved in both the diseases [75]. In fact, non-obese diabetic (NOD) mice fed with a GFD showed a lower incidence of diabetes, possibly also related to changes in the gut microbiome [76], and children with diabetes display a deranged immune response to gliadin [77], and the presence of TG2A deposits in the small intestinal mucosa [78]. This association represents a unique example of how our understanding of one disease improves knowledge and management of the other and *vice versa*.

### 4.2. Autoimmune Thyroid Disease

An increased prevalence of autoimmune thyroid diseases, namely Hashimoto’s and Graves’ diseases, has been described in adults and to a greater extent in children with CD, although the rate varies amongst studies [79,80,81,82]. In fact, when screening 90 children and adolescents with autoimmune thyroid diseases by using EMA, a rate of 7.7% of positivity was found [80], whilst when using the TG2A-IgA in a mixed population, a rate of 4.6% was observed, with only 2.3% of them having biopsy confirmed CD [82]. The decreased specificity of TG2A in this subset of patients compared to the general population was attributed to co-morbidities including type 1 diabetes and Down’s syndrome [82], even though the possibility that a considerable number of them suffer from potential CD cannot be ruled out. However, although CD seems to be more common in children with autoimmune thyroid diseases, the reverse is not necessarily true. A prospective study including 545 children and adolescents with CD on a GFD showed that autoimmune thyroid disease was no more common in these patients than in controls, and that patients who had been on a GFD longer were less likely to suffer from thyroid disorders [81]. Consequently, the authors suggest that screening CD children for autoimmune thyroid disorders should be performed only in those who are symptomatic or suspected of having this condition [81]. By contrast, when screening adolescents with CD for autoantibodies against thyroid peroxidase, a prevalence of 7.2% was observed in comparison with 2.8% found in controls [83]. On the other hand, the institution of a GFD did not prevent the progression of established autoimmune thyroid disease [84] or the appearance of anti-thyroid antibodies [85] after the diagnosis of CD. As in type 1 diabetes, a close overlap in the risk haplotypes between these conditions has been shown, with a frequent presence of HLA DQA1*0301 (linked to DR4), DQB1*0301 (linked to DR5), and DQB1*0201 (linked to DR3) [86].

### 4.3. Other Immune-Mediated Disorders

Other than type 1 diabetes and thyroid autoimmune diseases, further endocrine conditions, such as Addison’s disease [87] and primary hyperparathyroidism [88], together with a substantial number of additional immune-mediated disorders [89] have been reported in association with CD. These include rheumatic and connective tissue diseases, *i.e.*, Sjögren’s syndrome [90], systemic lupus erythematosus [91], and juvenile idiopathic arthritis [92], skin disorders [93], hepatic diseases [94], neurological abnormalities [95], cardiological illnesses, including autoimmune myocarditis [96] and idiopathic dilated cardiomyopathy [97], although a recent nationwide study found only a moderately but not statistically significantly increased risk of this latter condition in patients with biopsy-verified CD [98]. Similarly, the previously reported association with immunoglobulin (Ig)A mesangial nephropathy [99], has been recently contested [100]. Here below, we summarize the most frequent conditions associated with CD, while referring readers to the cited references for the others.

### 4.4. Rheumatic Disorders

Sjogren’s syndrome is an autoimmune exocrinopathy characterized by dry eyes, dry mouth, and circulating antibodies against intracellular proteins. The association between Sjogren’s syndrome and CD was first described in 1965 [101]. Since then, two further series reported prevalence rates of CD amongst adult Sjogren’s patients as high as 12% [102] and 14.4% [103]. With these values, Sjogren’s syndrome can be considered the most common rheumatic disorder associated with CD. In this regard, it should be emphasized that 56% of Sjogren’s adult patients carry the HLA-DQ2 haplotype [103]. The second most frequent rheumatic disorder associated with CD is antiphospholipid syndrome, with a prevalence of 14% in adults diagnosed with this condition [104]. The prevalence of CD amongst patients with chronic arthritis ranged from 1.5% to 2.5% [102,105,106], whilst its occurrence in patients with systemic lupus erythematosus is under debate, since despite reported findings of an increased rate [91,102], no case of CD was found in a large series of 103 systemic lupus erythematosus patients [107].

### 4.5. Selective IgA Deficiency

Selective IgA deficiency is one of the most frequent immunologic disorders associated with CD, with a reported frequency of 1:39 in populations of both adult and childhood CD [108], compared to an estimated frequency of 1:600 in the general population [109]. The reverse is also true, as there is an increased prevalence of CD found in children with IgA deficiency [110]. This close association may be largely due to a higher prevalence of the HLA-DQ2 genotype in the IgA deficient population too [111]. While IgA deficiency does not seem to affect CD presentation, it significantly impacts the diagnostic process since celiac antibodies utilized for the serological screening are primarily of the IgA subtype.

### 4.6. Neuro-Psychiatric Conditions

Other than gluten ataxia, a wide range of neurological and psychiatric disorders, such as peripheral neuropathy, epilepsy, headaches, dementia, depression, autism, and schizophrenia has been reported in association with CD, both in childhood and adulthood, although the risk seems higher in adulthood [95]. In particular, the syndrome of epilepsy with occipital calcifications was reported in childhood CD [112], although the strength of this association has recently been questioned, following evidence that only seven out 2893 epileptic children (0.2%) screened for CD presented with cerebral calcifications, possibly because cerebral calcifications might develop later in life [113]. Moreover, when considering the different types of epilepsy overall, the reported prevalence in CD is in line with that found in the general population [113]. The other neurological disorder of early childhood that has been associated with gluten ingestion, rather than with actual CD, is autism [114], stemming from the reported benefit of a gluten-free/casein-free diet in these patients [115]. Nevertheless, the association between autism and CD has not yet been conclusively proved [116]. Instead, there is some evidence of an increased prevalence of milder neurologic disorders in childhood CD, such as chronic headaches, hypotonia, attention deficit/hyperactivity disorder, and developmental delay [117]. Specifically, pediatric CD carries a three-fold increased relative risk of chronic headache, as compared to controls, a situation that improves dramatically after the implementation of a GFD [113].

### 4.7. Skin and Annexes

The relationship between dermatomyositis and CD has been suggested, both in young adult and adults [118,119], with some evidence of amelioration following a GFD [120]. Again, close association with DQA1*0501 heterodimer has been reported [121]. A similar benefit of GFD was shown also in a child with vitiligo [122], and in a few cases of alopecia areata associated with CD [123,124,125]. However, the scarcity of the cases described and the lack of systematic studies, do not allow for definite conclusions in skin disorders [93].

### 4.8. Liver

Possible conditions associated with CD, mainly in childhood, include elevated serum aminotransferases, without any specific histological changes that promptly reverse after a course of GFD, now known as “celiac hepatitis” [94], as well as autoimmune hepatitis, and primary sclerosing cholangitis. Primary biliary cirrhosis, whose diagnosis often precedes that of CD, has been found only in adults, while natural history is totally independent from the establishment of a GFD [94,126,127].

### 4.9. Reproduction

Obviously, this problem affects only CD in adulthood, where the association of infertility in both women and men [128] is recognized to such an extent that screening for CD is part of the routine diagnostic workup of infertile couples [129], also because the possibility of successful reproduction and pregnancy improves after diagnosis of CD and the start of a GFD [130]. Furthermore, celiac women also experience delayed menarche and early menopause more frequently than controls, thus leading to a shorter fertile life span [131]. Apparently, this finding was not confirmed in a recent study, showing that celiac women do not have a greater likelihood of clinically recorded fertility problems than non-celiac women do, even though this was not true in those diagnosed with CD between 25–39 years of age [132].

### 4.10. Genetic Disorders

Following the established association with some genetic disorders, namely Down’s, Turner, and Williams syndrome, CD screening is highly recommended for these conditions. The prevalence of CD in Down’s syndrome patients, indeed, ranges from 4% to 18%, according to several studies performed in both the USA and Europe [133,134,135], with a higher rate in adults than in children [136]. Whether it depends on a real increased prevalence with age or a missed diagnosis during childhood is unknown. CD has also been found to be associated with Turner syndrome, with reported prevalence ranging from 4.5% to 8.1% [137,138]. Finally, the association with Williams syndrome has been well documented, with an estimated prevalence of CD as high as 9.3% in a representative cohort of 63 patients, in an Italian multicenter study [139]. This relationship between genetic syndromes and CD does not seem to be dependent on a larger percentage of patients carrying the HLA-DQ2 or HLA-DQ8 haplotypes compared to the general population, but to a propensity to develop autoimmune associated diseases, including CD. For instance, a relationship with the pro-inflammatory cytokine interferon-α, which plays a key role in eliciting the intestinal immune response in celiac disease [16], has been suggested, since its receptor is encoded on chromosome 21 [140].

## 5. Response to GFD and Prognosis

**Figure 3:**
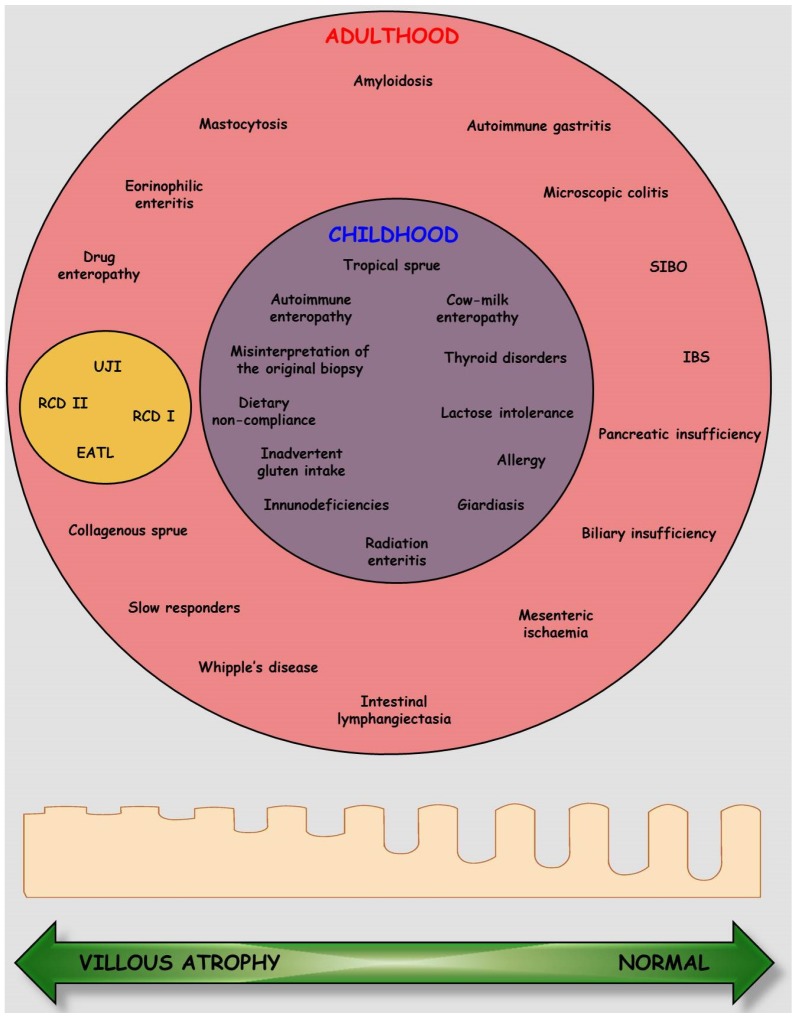
Possible causes of refractoriness to a gluten-free diet. A series of conditions responsible for refractoriness to gluten-free diet is presented according to their “true” (inside the yellow circle) or “false” relationship (all the others) with celiac disease, and to their prevalence in adulthood (**red circle**) or childhood (**blue circle**). At the bottom, a schematic representation of the duodenal mucosa architecture is given as a continuum (**green arrow**) from villus atrophy to a normal appearance, going from left to right, thus the place where each pathological condition is inserted in the circles depends on the status of the small intestinal mucosa. Abbreviations: EATL: enteropathy-type-associated T cell lymphoma; IBS: irritable bowel syndrome; RCD: refractory celiac disease; SIBO: small intestinal bacterial overgrowth; UJI: ulcerative jejunoileitis.

Pending a better understanding of its pathogenesis [1,2], the treatment of CD is still based on the GFD, as originally proposed by the Dutch Pediatrician, Doctor Willem-Karel Dicke [141], which requires the complete elimination from the diet of all types of foods containing or prepared with wheat, rye, barley grains, and their derivatives. Although this treatment guarantees the recovery from both clinical symptoms and intestinal damage in almost all cases, it seriously affects the patient’s quality of life, since its stringency and lifelong duration cause chronic distress and it segregates patients in a sort of “social apartheid” [142]. This is the reason why compliance is very often suboptimal, ranging from 52% to 95% in the pediatric population [143], with adolescents having serious difficulties with permanent adherence to the GFD [144]. In general, factors that improve chances of compliance are early age diagnosis [145], the presence of symptoms after gluten ingestion [146], a good awareness in the family [147], and frequent follow-ups by both a physician and a meticulous nutritionist [148]. Similarly, in adults the adherence to a GFD improves by having regular follow-ups, even by telephone, within the setting of a dedicated adult celiac unit [149]. Therefore, patients’ education, close supervision with scheduled nutritional counselling, and maintenance of dietary adherence when travelling or dining out, are all crucial factors needed to achieve full compliance [150]. This treatment carries the additional burden of a wide range of minor side effects, such as constipation, intestinal bloating, changes in body composition, modification of dietary intake, and poor vitamin status [151,152]. In fact, in a substantial proportion of adults, the presence of several nutritional problems has been found after a long-term course of GFD: calorie/protein imbalance, fiber, folate, niacin, vitamin B12, and riboflavin deficiencies [21]. In this regard, the nutritional adequacy of the GFD is particularly important in childhood, when maximum energy is required for growth, development and activity. In recent years, attention has been focused on the nutritional quality of the gluten-free products (GFPs) available on the market, and it has emerged that these are of lower quality and poorer nutritional value than their gluten-containing counterparts [74]. A further emerging problem is represented by excess weight and obesity [153,154], since 81% of patients on a GFD had gained weight after two years, including 82% of those who were initially overweight patients, in both adults and children [151]. Reasons for the weight gain are related to the improved intestinal absorption and the hypercaloric content of commercial or natural gluten-free food due to its high lipid and protein content, thus attentive follow-ups with an experienced dietitian is strongly recommended. Finally, in the last few years, a gap has emerged between the clinical and mucosal recovery, mainly in the adult population, since when re-biopsing treated CD patients only half of them had healed mucosa, despite the negativity of serologic markers [155,156]. This makes the follow-up of CD patients extremely difficult. In this regard, it has been recently shown that measuring the circulating levels of intestinal-fatty acid binding protein may provide a useful tool for monitoring compliance with a GFD [157]. Following the exclusion of gluten from the diet, the clinical symptoms and mucosal architecture usually improve very quickly in children [158], whilst in a mixed population, the histological response requires more time, reaching a complete recovery in 95% of cases within two years [159]. In fact, mostly in adulthood, the establishment of a strict GFD has intrinsic difficulties, from both a practical and psychological point of view. Moreover, both the clinical and mucosal recovery may be slower than expected in this age group, with as many as 60% of patients showing persistent villous atrophy after the same time course of two years on a GFD [160,161]. This is why a second biopsy is not required in childhood [39], whilst in adulthood it is the only tool that can detect an unsatisfactory histological response [162]. This indication arises also from the evidence that the persistence of intestinal damage carries an increased risk development of a malignant T-cell lymphoma [156,163], albeit not of overall mortality [164] and that the GFD does not fully protect patients from developing complications [156,163]. Since the latter are very rare conditions [163], the persistence or recurrence of symptoms should first prompt the clinicians to revise the accuracy of the original diagnosis and, if necessary, to perform a course of gluten challenge followed by new mucosal sampling [165]. Afterwards, it is advisable to carry out systematic evaluation for alternative and associated diseases, possibly responsible for the apparent refractoriness to GFD [166,167]. In this regard, false refractoriness may be divided on the basis of the mucosal architecture, in those conditions with a normal duodenal mucosa, and those causing complete or partial alteration of mucosal architecture which firstly include poor compliance to GFD, then a number of conditions, as shown in Figure 3.

### 5.1. Complicated Celiac Disease

The term complicated CD encompasses a spectrum of different conditions, namely refractory CD (RCD), ulcerative jejunoileitis (UJI), and enteropathy-type-associated T cell lymphoma (EATL), which represent a biological continuum and are estimated to affect only a few cases in the adult CD population [2,3,163]. The only two exceptions are an eight-year-old boy suffering from refractory CD, who was successfully treated with azathioprine [168], and a 13-year-old girl with CD and evidence of ulcerative lesions in the jejunum, who responded to a GFD [169]. However, in our opinion, the latter cannot be considered a true refractory case, firstly because of the response to a GFD and secondly for the absence of both a monoclonal re-arrangement of the T-cell receptor and an aberrant population of intraepithelial lymphocytes (IELs), which are the hallmarks of CD-associated UJI. Further causes capable of inducing ulcerative lesions in the small intestine should have been actively ruled out. On the other hand, since a systematic study with wireless capsule endoscopy or enteroscopy has not yet been carried out in the pediatric CD population, we cannot exclude the possibility that a number of erosions/ulcers may be present in pediatric active CD patients, devoid of the biological significance of a complication. In fact, the diagnostic criteria for the presence of refractoriness are very strictly defined as the persistence or recurrence of both malabsorption syndrome and intestinal villous atrophy after 6–12 months of a controlled GFD [32]. Over the last decade, two distinct conditions have been identified on the basis of the amount of aberrant IELs, named type I and type II [170]. The latter has a poor prognosis, largely due to the frequent development of an overt lymphoma [171]. By contrast, in type I RCD, intestinal lymphocytes have an almost normal phenotype, symptoms are usually mild and sometimes difficult to differentiate from uncomplicated celiac disease, except for the resistance to a GFD. Ulcerative jejunoileitis is characterized by the presence of several ulcerations possibly involving all layers of the intestinal wall, which may evolve in strictures, bleeding or perforation and often results in a life-threatening condition [172]. As a consequence, the main clinical features are colicky central abdominal pain, distension, fever, diarrhea, and weight loss. The current management of these conditions relies on a combination of nutritional support, immunosuppressive or biological therapies (steroids, azathioprine, cladribine, anti-CD52 or anti-CD30 monoclonal antibodies), and autologous hematopoietic stem cell transplantation whose choice is based on non-controlled studies in small cohorts of patients and personal experience [173]. However, their prognosis remains bleak. 

In conclusion, despite previous evidence that adult CD carries a twofold increase in all-cause mortality, and 60-fold increase in non-Hodgkin lymphoma compared with the general population [174], a recent competing risk analysis showed that overall, people with CD have no major excess risk of cancer, related digestive disease or respiratory disease or cardiovascular mortality compared with the general population, although an excess risk for non-Hodgkin lymphoma was confirmed [156,175]. Although these findings reassure both CD patients and clinicians involved in managing their care, it should be emphasised that the increased mortality risk is actually restricted to those diagnosed with CD after 40 years of age, where more vigorous follow-up is then recommended [176].

## 6. Conclusions

Taken together, these findings highlight how different CD in children appears compared to CD in adults. First, the disease seems more common in children than in adults [177,178] probably because of the frequent presence of malabsorption symptoms mainly in the early pediatric age. Secondly, coexisting autoimmune diseases are more common in adulthood, thus making the diagnosis in this age group more difficult. Thirdly, the CD-related complications, including the malignant forms, are almost exclusively found in adults. Finally, considering that this illness is a permanent intolerance to gluten, the relevance and intrinsic difficulty of the transition phase between these two periods of life deserves our utmost attention, since it represents a cornerstone in the modern management of chronic diseases. Transition, indeed, is recognised as not just a simple “transfer” of patients from the pediatric service to adult health care [179], but relies on a complex and scheduled process, aimed at ensuring continuity, coordination, flexibility and sensitivity in a multi-disciplinary context [180].
